# Evaluation of symptomatic small bowel stricture in Crohn’s disease by double-balloon endoscopy

**DOI:** 10.1186/s12876-023-02839-8

**Published:** 2023-07-20

**Authors:** Jing Hu, Juan Wu, Peipei Zhang, Naizhong Hu, Qiao Mei, Xingwang Wu, Wei Han

**Affiliations:** 1 Department of Gastroenterology, The First Affiliated Hospital of Anhui Medical University, No. 218 Jixi Road, Shushan District, Hefei, 230022 Anhui Province People’s Republic of China; 2 Department of Radiology, The First Affiliated Hospital of Anhui Medical University, Hefei, 230022 People’s Republic of China

**Keywords:** Double-balloon endoscopy, CT enterography, Crohn’s disease, Small bowel stricture

## Abstract

**Purpose:**

To assess the efficacy of double-balloon endoscopy (DBE) for the detection of small-bowel strictures in Crohn’s disease (CD).

**Methods:**

This tertiary-referral hospital cohort study was conducted between January 2018 and May 2022. CD patients with symptoms of small-bowel stricture were enrolled sequentially. All of the patients were subjected to both computed tomography enterography (CTE) and DBE, and their symptoms of stricture were assessed using the Crohn’s Disease Obstructive Score (CDOS). The diagnostic yield of DBE was compared to that of CTE, and the relationship between the DBE findings and CDOS was investigated. The factors influencing the DBE diagnosis were examined using Cox regression analysis.

**Results:**

This study included 165 CD patients. The CDOS scores were higher in 95 patients and lower in 70 patients. DBE detected 92.7% (153/165) and CTE detected 85.5% (141/165) of the strictures. The DBE diagnostic yields were 94.7% (90/95) in the high CDOS patients and 91.4% (64/70) in the low CDOS patients (P = 0.13). Patients with a history of abdominal surgery and abscess had a lower diagnosis rate in the multivariate analysis.

**Conclusion:**

DBE has been demonstrated to be an efficient diagnostic method for detecting small bowel strictures in CD patients. Additionally, there was no difference in the diagnostic yields between patients with low and high obstructive scores.

## Introduction

Crohn’s disease (CD) is a chronic inflammatory gastrointestinal disease that can lead to a variety of complications. Intestinal strictures are common CD complications, occurring in 15–30% of patients within the first 10 years after diagnosis [1, 2]. Strictures are frequently associated with obstructive symptoms, necessitating endoscopic and surgical intervention [3, 4]. Therefore, it is critical to accurately diagnose and evaluate CD stenosis.

Approximately 70–80% of CD lesions involve the small intestine, with 30% of the lesions being isolated small-bowel lesions [5–7]. Small-bowel CD has a more complicated pathology and is more likely to develop stricture complications than colonic CD [8–10].

Because of the unique anatomy and technical limitations, diagnosing and evaluating isolated small-bowel CD is difficult for gastroenterologists. Transabdominal ultrasonography (TUS), computed tomography enterography (CTE), magnetic resonance enterography (MRE), small-bowel capsule endoscopy (CE), and double-balloon enteroscopy (DBE) are some of the new endoscopic and radiologic techniques for evaluating small intestinal stenosis that have been developed in recent years (DBE).

TUS is a useful tool for the diagnosis and monitoring of small-bowel strictures [11]. However, accurate and dependable results in US depend on having seasoned operators. Numerous studies have reported on the detection efficiency and ability of MRE predict surgical outcomes [12, 13]. This method, however, is time-consuming and costly. Furthermore, the interobserver consistency of MRE has been variable [14]. CTE is extremely effective at detecting small-bowel CD, with a sensitivity of 83% and a specificity of 88% [15]. Additionally, the rapid collection and image reconstruction of CTE allows for visualization of the entire small bowel and extraintestinal lesions [16]. Although CE is a very useful noninvasive tool for evaluating intestinal mucosal lesions in CD patients with small-bowel involvement, capsule retention has been reported in up to 5–13% of patients with known Crohn’s disease [17–19]. The risk of capsule retention is much higher in patients with small-bowel obstruction [20]. Furthermore, tissue diagnosis and endoscopic treatment cannot be performed when necessary [21].

DBE has been developed in recent decades for treating small-bowel diseases [22, 23]. The benefits of this deep enteroscopy technique include more direct visualization of the small intestine, the ability to obtain tissue biopsies for histopathology, and the ability to treat strictures [24–26]. Hence, DBE has become a widely accepted modality for assessing small-bowel CD [27]. Previous studies assessed the efficacy of DBE for the diagnosis and treatment of CD, and the majority of these studies involved patients with isolated CD of the small bowel [28–31].

The relationship between CD small-bowel strictures detected by DBE and the severity of stenosis symptoms, however, remains unknown. In addition, the factors influencing the diagnosis of DBE in patients with small bowel CD are still unknown. Hence, we conducted a prospective cohort study to evaluate the diagnostic yield of DBE in small-bowel CD patients with a symptomatic stricture.

## Patients and methods

### Patients and data collection

**Fig. 1:**
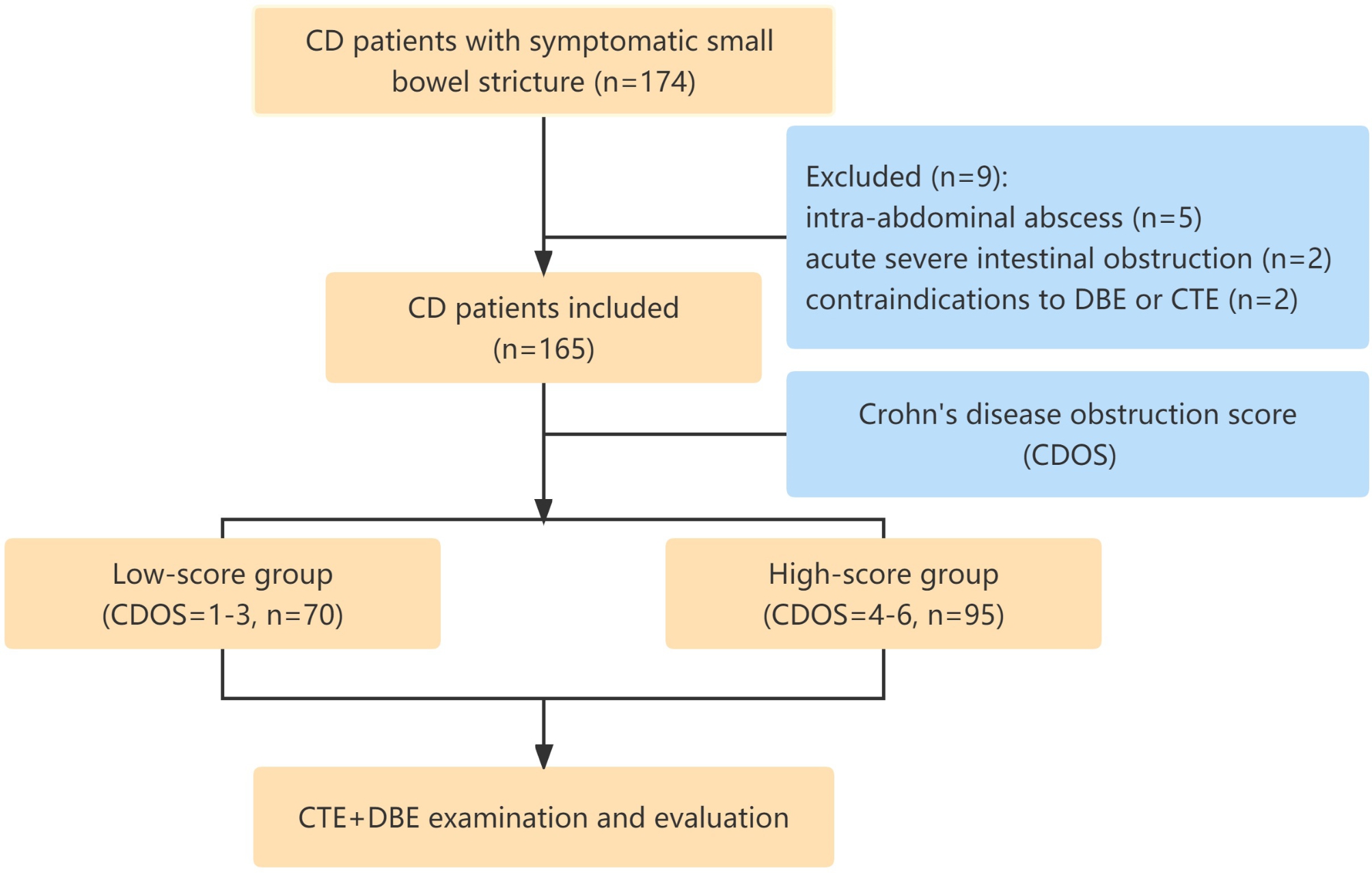
Diagnostic workflow of Crohn’s disease patients with symptomatic small-bowel stricture

From January 2018 to May 2022, 165 CD patients with symptomatic small bowel strictures were enrolled at Anhui Medical University’s First Affiliated Hospital. This facility is a tertiary care facility for inflammatory bowel disease (IBD). All of the included patients met the following inclusion criteria: (1) a defined diagnosis of CD; (2) small bowel stricture symptoms; and (3) isolated small bowel strictures. Patients with an intra-abdominal abscess, suspected perforation, acute strangulated intestinal obstruction, contrast media allergies, or contraindications to DBE or CTE were excluded. All of these patients underwent CTE and DBE and were preoperatively followed up (Fig. 1). The time between CTE and DBE was reduced to less than one month. For the DBE procedures, all patients provided informed consent.

The prospectively obtained demographic and clinical data included sex, age, time from CD diagnosis to DBE, location of CD, previous surgery, laboratory values (complete blood cell count, albumin, C-reactive protein), and CD activity index [32].

### The severity of stenosis symptoms

The Crohn’s Disease Obstructive Score (CDOS) was used to assess and quantify obstructive symptoms. The score was developed based on four core items (obstructive pain feature, signs of nausea, vomiting, dietary restriction, and hospitalization) and was tested in a recent clinical study [33]. In the CDOS, the severity of stenosis symptoms is graded from 1 to 6. The patients were divided into low- (1–3) and high-score (4–6) groups to compare the relationship between the DBE findings and obstructive severity.

### DBE procedure and evaluation

All patients who underwent DBE procedures were sedated with a combination of intravenous and inhalation anesthesia. DBE was performed with an EN-580T enteroscope (FUJIFILM, Tokyo, Japan) and an overture, which was performed by three IBD endoscopists with at least 200 cases of experience. The insertion route was chosen according to the estimated location of the suspected lesion, mainly based on the results of CE or radiological findings (i.e., enteroclysis, CTE or MRE ). If the location of the small-bowel lesion is unknown or uncertain, clinical presentation of small bowel stricture was the basis for starting DBE from antegrade or retrograde approach. Oral administration of 2000 ml polyethylene glycol-electrolyte lavage solution (Beaufour Ipsen Industrie, Dreux, France) 4 h before the retrograde DBE examination was used for bowel preparation. Antegrade DBE was performed after an 8-hour fast and before processing.

The depth of DBE insertion was calculated using a method described in the previous literature [34]. CD-associated DBE stricture was defined as failure to pass the endoscope or an internal diameter of the small-bowel lumen of less than 10 mm [35]. During the DBE examination, the small-bowel stricture site was described as either the jejunum, terminal ileum, or proximal ileum [36]. The jejunum was defined as the section of the small bowel from the proximal part of the small bowel to the proximal part of the ileum. The terminal ileum was defined as a 10 cm section from the ileocecal valve. The proximal part of the ileum was defined as the section of the bowel between the proximal end of the terminal part and the terminal ileum.

### CT enterography procedure and evaluation

Four hours before CTE examinations, all patients underwent intestinal preparation with 2000 ml polyethylene glycol-electrolyte lavage solution (Beaufour Ipsen Industrie, Dreux, France). Before scanning, 1500 ml of mannitol solution was taken orally, eventually reaching the small bowel for evaluation. A 128-slice MDCT scanner was used to perform CTE (GE Medical System, Chicago, IL, USA). The scan parameters were as follows: 5 mm layer thickness and spacing; 1.375:1 pitch; kV 120; and mAs 300. After injecting 100 ml of contrast agent (320 mgI/mL) into the elbow vein, the entire abdomen was scanned with delays of 45 and 90 s. An experienced gastrointestinal radiologist who was blinded to the clinical and endoscopic information analyzed the CTE images.

On CTE imaging, intestinal strictures were defined as follows: a localized thickened bowel wall and constriction of the intestinal lumen, enhanced bowel wall thickness ≥ 25%, reduction in luminal diameter ≥ 50%, and dilation of the small intestine proximal to the stricture ≥ 3 cm [37].

### Statistics

Means ± SDs or medians and range are used to describe quantitative variables. The DBE and CTE diagnostic yields were expressed as percentages and compared using the chi-square test. The factors influencing the DBE diagnosis were examined using Cox regression analysis. Variables with P < 0.05 in the univariate analysis were tested further in a multivariate analysis. SPSS 21.0 was used for all statistical analyses (IBM Corporation, Armonk, NY, USA). P < 0.05 was regarded as statistically significant.

## Results

### Patient characteristics and severity of stricture symptoms at baseline

A total of 174 patients with symptomatic small intestinal strictures were enrolled in the study from January 2018 to May 2022. Nine patients were excluded due to intra-abdominal abscess (n = 5), acute severe intestinal obstruction (n = 2), or contraindications to DBE or CTE (n = 2). Hence, this study included 165 CD patients with small-bowel strictures (Fig. 1). Table 1 shows the baseline patient characteristics. According to the Montreal classification [38], all patients had a stricture phenotype. Previous small intestinal surgery was linked with CD in 41 (24.8%) patients. Forty-two (25.5%) of the patients had a previous intra-abdominal abscess or an intestinal fistula but not at the time of enrollment. The patients were divided into two groups based on the severity of their stricture symptoms. The low-score group was comprised of 70 (42.4%) patients with CDOS = 1–3 (16 patients, CDOS = 1; 18 patients, CDOS = 2; 36 patients, CDOS = 3), while the high-score group was comprised of 95 (57.5%) patients with CDOS = 4–6 (45 patients, CDOS = 4; 36 patients, CDOS = 5; 14 patients, CDOS = 6).

**Table 1:** Clinical characteristics of patients included in this study

Variable	Characteristic
Gender, N (female/male)	41/124
Age at diagnosis, years (median, range)	33 (15–66)
Disease duration, months (median, range)	24 (1–240)
Previous abdominal surgery, N (%)	41 (24.8)
Previous intra-abdominal abscess/intestinal fistula, N (%)	42 (25.5)
Crohn’s disease phenotype, N(%)	
L1 (ileal disease) L2 (colonic disease) L3 (ileocolonic disease) L4 B1 (nonpenetrating/stricturing) B2 (stricturing disease) B3 (penetrating disease) P (Perianal disease)	110 (66.7) 0 (0) 55 (33.3) 42 (25.5) 0 (0) 165 (100) 0 (0) 47 (28.5)
Hb, g/L (mean ± SD)	121.16 ± 21.42
Alb, g/L (mean ± SD)	37.24 ± 4.72
CRP, mg/L (mean ± SD)	13.95 ± 7.72
CDAI (median, range)	317 (125–459)
CDOS, N (low/high score)	70/95

Hb, hemoglobin; Alb, albumin; CRP, C-reactive protein; CDAI, CD activity index; CODS, Crohn’s Disease Obstructive Score

### Results of DBE and CTE

In our study, 165 CD patients with symptomatic small intestinal strictures underwent 179 DBEs. The antegrade route was used for 14 procedures, while the retrograde route was used for 137 procedures. DBE was performed via both routes on 14 patients. In the DBE procedures, no patient experienced adverse events (such as anesthesia accident, gastrointestinal perforation or hemorrhage, or pancreatitis).

In the antegrade DBE examinations, the mean length of insertion was 237.14 ± 88.61 cm, and in the retrograde procedure, it was 99.83 ± 60.13 cm. DBE detected 168 strictures. In 136 patients, there was a single stricture. DBE detected small-bowel strictures in the jejunum (23, 13.7%), proximal ileum (131, 78.0%), and terminal ileum (14, 8.3%).

The overall diagnostic yield of DBE in CD patients with small-bowel strictures was 92.7% (153/165 patients), while with CTE, it was 85.5% (141/165 patients). DBE and CTE both detected strictures in 137 patients. Sixteen patients had DBE-positive strictures but negative CTE results. Of these, 15 had disease restricted to the ileum, and 1 had disease in the jejunum. Strictures were not accessible at DBE in 4 cases, which resulted from adhesions because of previous intra-abdominal abscess/intestinal fistula and history of CD-associated abdominal surgery, but were all detected by CTE.

We then associated the DBE or CTE findings with the severity of stricture symptoms. Based on the CDOS, the patients were divided into the low-score and high-score groups. The DBE diagnostic yields were 91.4% and 94.7% in the low-score and high-score groups, respectively (P = 0.13). Intriguingly, patients in the high-score group had a significantly higher CTE diagnostic yield than those in the low-score group (90.1% vs. 75.9%, P = 0.01).

Over the course of DBEs, 10 strictures in 6 patients were dilated. Obstructive symptoms were relieved after balloon dilatation in all patients. Within the study period, 5 of 6 patients remained surgery-free. In terms of surgery, stricturoplasty and bowel resection were performed in 3 and 5 patients, respectively.

### Factors associated with successful detection of DBE

Univariate analysis was used to examine the factors associated with successful detection using DBE (gender, age of diagnosis, disease duration, history of CD-associated abdominal surgery, previous intra-abdominal abscess/intestinal fistula, levels of CRP, CDAI, location of disease, perianal disease, severity of stricture symptoms). Variables with P < 0.05 in the univariate analysis were tested further in a multivariate analysis. In the multivariate analysis, previous intra-abdominal abscess/intestinal fistula (hazard ratio = 2.021, 95% confidence interval (CI): 1.075–3.826, P = 0.021) and history of CD-associated abdominal surgery (hazard ratio = 2.852, 95% CI: 1.146–3.467, P = 0.017) were considered independent prognostic factors (Table 2).

**Table 2:** Factors associated with the efficacy of DBE for all patients

All patients (N = 165)	Univariate HR (95% CI), p-value	Multivariate HR (95% CI), p-value
Male	1.131 (0.709–1.632), 0.412	--
Age at diagnosis (years)	0.878 (0.862–1.003), 0.372	--
Disease duration (months)	1.015 (0.985–1.073), 0.613	--
Previous abdominal surgery	2.031 (1.257–3.643), 0.003	2.852 (1.146–3.467), 0.017
Previous intra-abdominal abscess/intestinal fistula	2.271 (1.239–4.285), 0.006	2.021 (1.075–3.826), 0.021
Disease location (Ileal)	1.419 (0.814–1.793), 0.220	--
Perianal disease	0.859 (0.592–1.256), 0.495	--
Clinical activity (CDAI>150)	1.031 (0.798–1.235), 0.650	--
Severity of stenotic symptoms (high-CODS)	1.403 (1.009–2.276), 0.075	--
CRP positive (>10 mg/L)	1.371 (0.872–2.023), 0.197	--

HR hazard ratio, CI confidence interval

## Discussion

In this study, we evaluated the efficacy and safety of DBE for detecting small-bowel strictures in CD patients. Our study’s main findings were as follows: (1) DBE was an effective method for diagnosing strictures in CD patients with obstructive symptoms. (2) The severity of stricture symptoms did not affect the diagnostic yield of DBE. (3) A history of abdominal surgery and abscess was linked to the failure to detect DBE.

Up to 67% of CD cases involve the small bowel [39], with 10–30% of cases involving solitary lesions in the small bowel [40]. Small-bowel strictures in CD patients can be difficult to diagnose, particularly in patients with extensive small-bowel involvement. The DBE technique has made examination of the entire small-bowel feasible [41], let alone the investigation of the deep small bowel [22].

DBE is an efficacious tool for evaluating small-bowel strictures linked with CD, according to our findings. The diagnostic yield of DBE procedures performed by experienced IBD endoscopists was 92.7%. Several studies found that the diagnostic yield of DBE in CD patients ranged from 22 to 70% [26, 42–44]. However, in a study comparing the diagnostic yields of DBE and fluoroscopic enterolysis, Naoki Ohmiya et al. found that DBE had a diagnostic yield of up to 95% for small-bowel obstruction [45]. A comparison study compared MR and balloon enteroscopy for small-bowel strictures in CD [13]. All strictures in 57 patients that were detected by balloon enteroscopy were MR-positive. Furthermore, 37 patients had endoscopic strictures that could not be detected using MR imaging. The following are some possible explanations for our study’s high diagnostic yield: (1) All DBE procedures were performed by IBD endoscopists with at least 200 cases of experience. (2) Based on previous medical history, either antegrade or retrograde DBE was chosen. The patients in this study had symptomatic small-bowel strictures and did not have early-stage disease.

DBE complications have been reported in determining the safety of this technique for assessing the small bowel in CD patients. According to these findings, the rate of complications (e.g., bleeding, perforation, and pancreatitis) ranged between 1.2% and 1.6% [46, 47]. Nonetheless, no DBE-related adverse events were observed during our diagnostic process, confirming the safety of DBE even in CD patients with small bowel strictures.

Previous research has shown that CTE and MRE are both valuable diagnostic techniques for investigating small-bowel lesions in CD patients [48, 49]. MRE has the benefits of no requirement for radiation exposure and includes high temporal and spatial resolution. However, CTE outperforms MRE in terms of scan time, lack of artifacts, and availability in most hospitals [50]. Consequently, CTE has been recommended as a useful tool for assessing disease activity and complications in CD involving the small bowel [51, 52]. Abnormal CTE results in the small bowel normally indicate the need for DBE, so it is critical to compare DBE and CTE findings. In our study, we compared the diagnostic yields of DBE and CTE in patients with small-bowel obstructive symptoms, and DBE correctly detected more strictures than CTE. An early study looked at the role of CT in the diagnosis of small intestine obstruction. CT results were used to correctly identify 63% (29 of 46) of those with small-bowel obstruction [53]. CTE outperforms conventional CT in detecting small-bowel strictures. When different criteria and gold standards are used in different studies, the sensitivity of CTE for the detection of small-bowel stenosis ranges from 85 to 93% [28, 54–56]. In this study, we found that the overall diagnostic yield of CTE for establishing a diagnosis of small bowel obstruction was 85.5%, which is consistent with previous findings. Although CTE’s diagnostic ability was found to be equivalent to DBE’s, we discovered that the diagnostic efficacy varied according to the severity of symptoms. Maglinte et al. classified patients with small intestine obstruction into low-grade and high-grade partial obstructions, with CT detecting 81% of the high-grade obstructions and 48% of the low-grade obstructions [53]. These findings could be attributed to DBE’s ability to directly visualize mucosal lesions.

Our study examined not only the diagnostic ability of DBE in CD patients with small-bowel strictures but also the factors associated with DBE efficacy in these patients. Previous intra-abdominal abscess/intestinal fistula and history of CD-associated abdominal surgery were considered independent prognostic factors of DBE detection failure. Adhesions from previous surgeries and a complicated phenotype (such as intra-abdominal abscess or fistula) of CD may make DBE insertion difficult. In a multicenter retrospective study investigating DBE results and the influence on CD management, the target area of 17% of patients could not be reached due to adhesions from previous surgeries, which limited deep penetration [27]. Kohei Matsushita et al. investigated the efficacy and safety of DBE in pediatric patients after surgery. In four postoperative patients and 2 nonoperative patients, there was difficultly in transanal pleating due to adhesions or thickening of the intestinal wall caused by inflammation (P = 0.02) [57]. These findings are consistent with our conclusion that DBE has limitations due to strongly adhered adhesions in CD patients.

There were several potential limitations to this study. First, this was a single-center study. The patients in the study were all enrolled at a tertiary care facility. Second, all DBE procedures were conducted by three experienced IBD endoscopists, which may result in a higher diagnosis rate and better outcomes. Finally, further clinical outcome analysis in CD patients with small bowel strictures should be conducted.

Our study concludes that DBE is an effective and safe method for assessing CD patients with small-bowel strictures. Furthermore, the benefit of DBE was demonstrated in low-grade obstructions. Previous intra-abdominal abscess/intestinal fistula and history of CD-associated abdominal surgery were considered independent prognostic factors of DBE detection failure.

## Data Availability

Available data are all presents in the paper.
